# Endoscopic ultrasound-guided emergency choledochoduodenostomy through a double duodenal stent

**DOI:** 10.1055/a-2307-5889

**Published:** 2024-05-07

**Authors:** Marco Sacco, Ludovica Dottori, Maria Teresa Staiano, Stefania Caronna, Silvia Gaia, Giorgio Maria Saracco, Mauro Bruno

**Affiliations:** 1 18691 Gastroenterology Unit, Azienda Ospedaliero Universitaria Città della Salute e della Scienza di Torino, Turin, Italy; 2 Department of Medical-Surgical Sciences and Translational Medicine, SantʼAndrea Hospital, Sapienza University of Rome, Rome, Italy

With the advancement of oncologic and endoscopic therapies, the survival of patients with pancreatic cancer is increasing, even in patients with advanced disease, meaning complications due to previous treatments are being seen more frequently. We describe the case of a 57-year-old woman with advanced pancreatic adenocarcinoma that had been diagnosed 2 years before admission who presented with jaundice requiring biliary stenting. She had developed gastric outlet obstruction 1 year after diagnosis and an initial duodenal uncovered self-expandable metal stent (USEMS) had been placed, which was then followed by placement of a second stent because of tumor ingrowth ( Fig. 1 ).

**Fig. 1:**
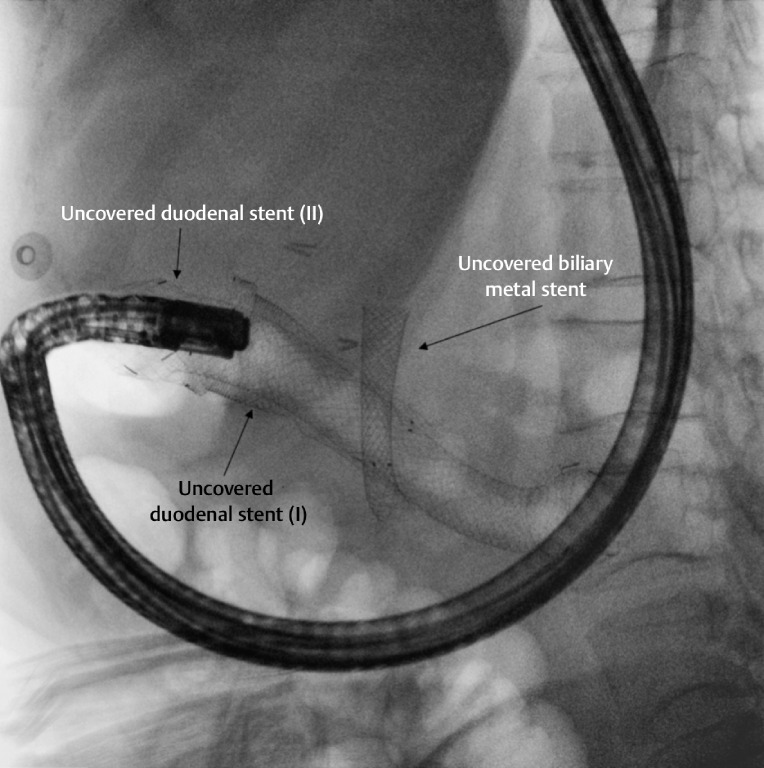
Fluoroscopic image during attempted endoscopic retrograde cholangiopancreatography with a duodenoscope showing the two previously placed duodenal self-expandable metal stents and a biliary stent in position.

The patient came to our attention, a few months after placement of the second duodenal USEMS, because of acute cholangitis due to blockage of the biliary USEMS (white cell count 36.5 × 10 ^9^ /L, total bilirubin 14.8 mg/dL, C-reactive protein 229.2 mg/dL). An endoscopic retrograde cholangiopancreatography was attempted, but it was not possible to recognize either the major papilla or the biliary stent, and an endoscopic ultrasound-guided biliary drainage (EUS-BD) procedure was therefore planned ( Video 1 ).

Endoscopic ultrasound-guided choledochoduodenostomy is performed through a double metal duodenal stent.Video 1

On EUS, the only visible window for biliary drainage was through the meshes of the duodenal stents, where the common bile duct appeared to be dilated up to 18 mm. We performed a choledochoduodenostomy with an electrocautery-enhanced lumen-apposing metal stent (LAMS) delivery system (Hot Axios; 6 × 8 mm; Boston Scientific) ( Fig. 2 ). After deployment, purulent bile flowed through the stent and correct positioning of the LAMS was verified with fluoroscopy ( Fig. 3 ). The procedure was uncomplicated. The patient gradually improved both clinically and biochemically, was able to resume oral feeding, and was discharged to a hospice after 10 days.

**Fig. 2:**
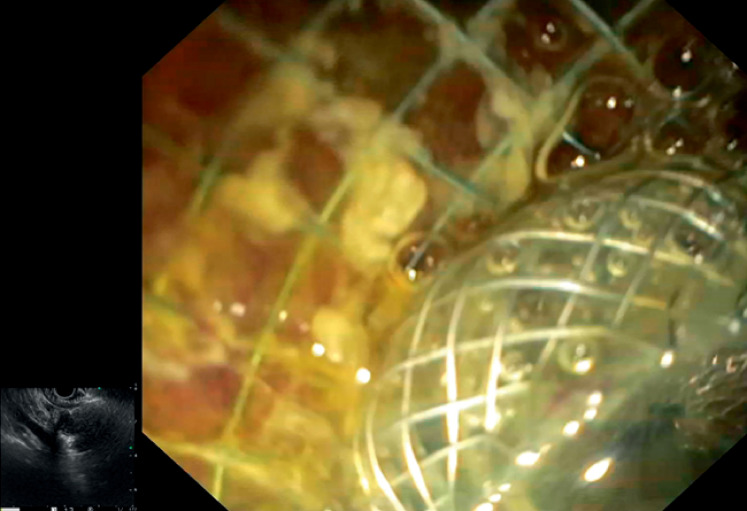
Endoscopic image showing the lumen-apposing metal stent deployed through the duodenal stent meshes.

**Fig. 3:**
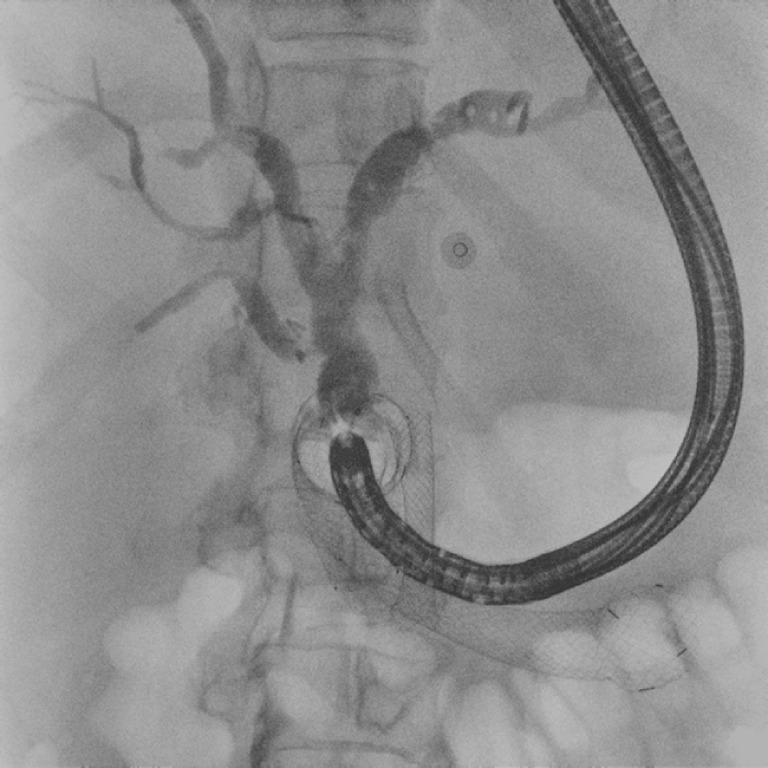
Fluoroscopic image during cholangiography showing correct functioning of the choledochoduodenostomy.

To our knowledge, this is the first report of successful EUS-BD through a double duodenal SEMS. This case again shows that improvements in, and the increasing spread of, interventional EUS skills allow the safe management of complications, which are being seen more frequently owing to longer life expectancy, in patients with pancreatic cancer, even where previous biliary or duodenal stenting has been performed, as is being increasingly commonly described 1 2 3 4 .

Endoscopy_UCTN_Code_TTT_1AO_2AG_3AZ

## Notes

Endoscopy E-Videos https://eref.thieme.de/e-videos
*E-Videos* is an open access online section of the journal *Endoscopy* , reporting on interesting cases and new techniques in gastroenterological endoscopy. All papers include a high-quality video and are published with a Creative Commons CC-BY license. Endoscopy E-Videos qualify for HINARI discounts and waivers and eligibility is automatically checked during the submission process. We grant 100% waivers to articles whose corresponding authors are based in Group A countries and 50% waivers to those who are based in Group B countries as classified by Research4Life (see: https://www.research4life.org/access/eligibility/ ). This section has its own submission website at https://mc.manuscriptcentral.com/e-videos .
